# Combating resistant pathogens: exploring the efficacy of eravacycline utilization in multidrug-resistant infections

**DOI:** 10.1128/spectrum.01314-25

**Published:** 2025-11-06

**Authors:** Kiara Patino, Ryan Vathy, Ben Albrecht, Sujit Suchindran, Sarah B. Green

**Affiliations:** 1 Department of Pharmacy, Emory Healthcare 14360, Atlanta, Georgia, USA; 2 Emory University School of Medicine 12239, Atlanta, Georgia, USA; 3 Division of Infectious Diseases, Emory University School of Medicine 12239, Atlanta, Georgia, USA; Houston Methodist, Houston, Texas, USA

**Keywords:** eravacycline, multidrug resistance, antimicrobial resistance, antibiotic resistance, *Stenotrophomonas*, VRE, CRE, MDR

## Abstract

Eravacycline, FDA-approved for complicated intra-abdominal infections, is increasingly used off-label for multidrug-resistant infections, including carbapenem-resistant Enterobacterales, *Stenotrophomonas maltophilia,* and carbapenem-resistant *Acinetobacter baumannii*. This retrospective, real-world study aims to evaluate the efficacy and safety of eravacycline for treating multidrug-resistant infections at an academic-based health system in Atlanta, Georgia. A multi-center, retrospective study was conducted on adult patients (≥18 years) who received eravacycline at a 6-hospital health system in Atlanta, Georgia, from 1 October 2022 to 30 June 2024. Patients treated for infections due to vancomycin-resistant Enterococcus, carbapenem-resistant Enterobacterales, carbapenem-resistant *Acinetobacter baumannii*, multidrug-resistant Enterobacterales, or *Stenotrophomonas maltophilia* were included. The primary endpoint was a composite of treatment failure, defined as inpatient mortality and/or 30-day microbiologic recurrence. Secondary outcomes included safety assessments for adverse events and *Clostridioides difficile* infection. Multivariate regression was used to identify risk factors for treatment failure. Forty-eight patients were included in this study. The patients’ median age was 57.6 years, with 60.4% female and 54.2% Black. Eravacycline was primarily used for vancomycin-resistant Enterococcus (31.3%), *Stenotrophomonas maltophilia* (29.2%), and carbapenem-resistant Enterobacterales (20.8%) infections. The median treatment duration with eravacycline was 12.6 days. Treatment failure occurred in 39.6% of patients, while 60.4% of patients experienced survival without recurrence. Adverse events were rare (4.2%). No significant factors for treatment failure were identified in multivariate analysis due to a lack of association. Eravacycline is a well-tolerated option for treating multidrug-resistant infections. Given its favorable safety profile, eravacycline is a promising alternative for infections with limited treatment options.

**IMPORTANCE:**

The increasing prevalence of multidrug-resistant infections, including carbapenem-resistant Enterobacterales and vancomycin-resistant Enterococcus, has made successful treatment of infections due to these organisms challenging. This study contributes to the growing body of literature on the use of eravacycline for multidrug-resistant infections, offering evidence of its safety and efficacy in this context. However, current guidelines do not widely endorse the use of eravacycline due to limited data despite its potential advantages. Many guideline-recommended treatments for these organisms have significant drawbacks, including substantial toxicity, collateral damage such as resistance selection, increased risk of *Clostridioides difficile* infection, and higher healthcare costs. Eravacycline may serve as a valuable salvage therapy in such scenarios due to its broad-spectrum activity and lower *Clostridioides difficile* risk. As resistance patterns evolve, eravacycline’s broad-spectrum activity may become a more used option in the management of difficult-to-treat infections, expanding therapeutic options for patients with few viable antimicrobial therapies.

## INTRODUCTION

The prevalence of multidrug-resistant (MDR) infections has been steadily increasing, posing a significant public health challenge including increased morbidity and mortality (1). According to the Centers for Disease Control and Prevention (CDC), antimicrobial resistance (AMR) threats, particularly those associated with carbapenem-resistant Enterobacterales (CRE) and vancomycin-resistant Enterococcus (VRE), have escalated since 2019 (1). These resistant pathogens present a critical concern due to the limited therapeutic options available, highlighting an urgent need for new and effective treatments. Eravacycline is a synthetic fluorocycline antibacterial agent FDA approved in 2018 for patients ≥ 18 years of age for complicated intra-abdominal infections (cIAIs) (2). Eravacycline has unique modifications in the tetracycline ring at C7 and C9 which assist in combating traditional tetracycline-acquired resistance mechanisms of efflux pumps and ribosomal protein mutations (3). Eravacycline’s broad spectrum of activity of Gram-positive, Gram-negative, anaerobic, and atypical pathogens makes it an appealing agent for MDR infections with little to no treatment options such as CRE, *Stenotrophomonas maltophilia* (*S. maltophilia*), carbapenem-resistant *Acinetobacter baumannii* (CRAB) VRE, and other MDR infections (4, 5). Compared to tigecycline, eravacycline has also been shown to have higher microbiological response rates and 2- to 8-fold lower MICs against multi-drug-resistant Gram-negative pathogens (6). The clinical significance of these differences remains unclear due to variations in the pharmacokinetic profiles of the two agents (7).

Despite the promising activity of eravacycline, the Infectious Diseases Society of America (IDSA) 2024 Guidance on the Treatment of Antimicrobial Resistant Gram-Negative Infections does not support the routine use of eravacycline for MDR infections, primarily due to the limited clinical data available (7). Instead, eravacycline is only recommended if other agents are not well tolerated or active (7). Specifically, for CRE, tigecycline and eravacycline are alternative treatments for infections that do not involve the bloodstream or urinary tract. Their effectiveness is not affected by the presence or type of carbapenemase (7). This recommendation is informed by an observational study showing that tetracycline derivatives have been associated with increased mortality in patients with urinary and bloodstream infections (8). Much of the evidence for this guidance comes from studies on tigecycline, with findings extrapolated to eravacycline due to the limited data available for this newer agent.

For the treatment of CRAB infections, the IDSA guidelines suggest that high-dose minocycline or high-dose tigecycline may be included as part of combination therapy (7). Clinical trials assessing the efficacy of eravacycline included only a small number of patients with CRAB infections, and there are limited post-marketing reports available to further establish its effectiveness (7). One observational study found that 23 patients treated with eravacycline experienced longer durations of mechanical ventilation and increased 30-day mortality (9). Additionally, all four patients with CRAB bloodstream infections treated with eravacycline died (9). Given the limited clinical data supporting its use, the guidelines recommend reserving eravacycline for cases where other treatments are ineffective, poorly tolerated, or unavailable (7).

For *S. maltophilia* infections, the guidelines suggest high-dose minocycline as a viable treatment option when used in combination therapy (7). Minocycline is preferred over tigecycline due to its slightly superior *in vitro* efficacy, more favorable pharmacokinetic and pharmacodynamic properties, availability in an oral form, and potentially better tolerability (7). In contrast, there is limited *in vitro* and *in vivo* evidence supporting the effectiveness of eravacycline against *S. maltophilia* (7).

Not included in the 2024, IDSA AMR guidance was a retrospective, observational, multi-center study published in 2023 that looked at eravacycline administration data from the previous 4 years from 19 medical centers. Overall, the study included 416 patients treated with at least 3 days of eravacycline with 75.7% achieving treatment success, defined as survival and absence of microbiologic recurrence within 30 days (5). The study included many patients with infections due to MDR organisms including CRE (10.3%, *n* = 43/416), CRAB (11%, *n* = 46/416), *S. maltophilia* (9.9%, *n* = 41/416), and VRE (11.8%, *n* = 49/416) (5). This study was the largest report of eravacycline utilization to date but still warrants further research on the safety and efficacy of eravacycline for the treatment of complicated MDR infections.

Eravacycline is a well-tolerated antimicrobial, with the most commonly reported adverse effects being infusion site reactions, diarrhea, nausea, and vomiting, while a study by Kunz-Coyne and colleagues found that approximately 9.4% of 416 patients experienced adverse effects, primarily gastrointestinal intolerance and hepatotoxicity (3, 5). Eravacycline also has a much lower rate of discontinuation and better tolerability than tigecycline, with studies demonstrating a significantly superior microbiological response rate compared to tigecycline (6). This favorable safety profile is particularly notable given the significant toxicities associated with many of the IDSA-preferred agents for treating these infections. For instance, ceftazidime-avibactam has been linked to the emergence of resistance following exposure, raising concerns about its long-term efficacy (7, 10). Meropenem-vaborbactam and imipenem-cilastatin-relebactam, while effective against CRE, carry risks of nephrotoxicity, hypersensitivity reactions, and *Clostridioides difficile* infection (CDI) (7, 11). Cefiderocol, a siderophore cephalosporin, has been associated with increased all-cause mortality in critically ill patients with multidrug-resistant infections, along with adverse effects such as diarrhea, infusion site reactions, and liver enzyme abnormalities (7, 12). Sulbactam-durlobactam, a key agent for treating CRAB, has been linked to elevated liver enzymes, hypokalemia, anemia, and thrombocytopenia, with a substantial cost burden of $530.21 per 0.5 g/1 g dose (13, 14). These toxicities highlight the need for alternative treatment options with improved safety profiles, such as eravacycline, particularly in patients who may not tolerate first-line agents.

Eravacycline has been utilized at this academic-based, 6-hospital health system in Atlanta, Georgia, for MDR infections with limited treatment options including CRE, *S. maltophilia*, and CRAB. The objective of this study is to evaluate the efficacy and safety of eravacycline utilization for off label treatment of infections caused by MDR organisms at the health system.

## MATERIALS AND METHODS

This study is an institutional review board-approved, multi-center, retrospective analysis of patients who received eravacycline at a 6-hospital health system from 1 October 2022 to 30 June 2024. These hospitals encompass both academic and university-affiliated centers, as well as community hospitals, located in urban and suburban areas in the Metro-Atlanta area. Patients with eravacycline administrations during the study time period were accessed through the health system’s electronic medical record, EPIC, SlicerDicer medication administration report. Patient charts were manually reviewed through EPIC.

The initial data inquiry identified 161 patients with eravacycline administrations. Adult patients, ≥18 years of age, were included if they received ≥72 h of treatment with eravacycline for the treatment of infections due to VRE, CRAB, CRE, MDR Enterobacterales, or *S. maltophilia*. MDR Enterobacterales was defined as non-susceptibility to at least one agent in three or more antimicrobial categories. Data were collected by performing a manual chart review for the included patients to collect baseline characteristics, clinical course, as well as primary and secondary outcomes.

The primary endpoint was a composite of treatment failure defined as inpatient mortality during the index admission and/or 30-day microbiologic recurrence. Microbiologic recurrence was defined as a positive culture for the same organism at any culture site within 30 days following the completion of eravacycline therapy. Secondary safety outcomes included incidence of antibiotic-associated adverse effects, infusion site reactions, and CDI diagnosis within 90 days of eravacycline administration. Baseline demographics included concomitant antimicrobial therapy, defined as any antimicrobial treatment initiated within 72 h of starting eravacycline. Patients were classified as requiring vasopressors or mechanical ventilation if they received either intervention within 24 h of the index culture. Intensive care unit (ICU) admission was determined based on the patient’s status at the time of the index culture. The Charlson Comorbidity Index was used to assess the patients’ overall disease burden. Immunocompromised status was defined using the CDC’s definition of immunocompromised status, which includes as having undergone bone marrow transplantation (BMT) or solid organ transplant within the past 12 months, chemotherapy within the past 6 months, Human Immunodeficiency Virus (HIV) with a CD4 count <200, or the use of immunomodulatory agents within the past 6 months. Immunomodulatory agents included antimetabolites, alkylating agents, immunosuppressive medications for transplants, Tumor Necrosis Factor (TNF) inhibitors, prednisone (20 mg or more and equivalents per day for >14 days), and monoclonal antibodies for autoimmune diseases (15).

Descriptive statistics were used to evaluate baseline characteristics, clinical course, and the primary and secondary outcomes. Discrete data were reported as frequencies and percentages, while continuous data were summarized using either the median and interquartile range (IQR) or the mean and standard deviation (SD), depending on the distribution’s normality. A multivariate regression analysis was done using IBM SPSS (Statistical Package for Social Science) ver. 29 (Armonk, NY: IBM Corp) to assess risk factors for treatment failure with eravacycline. The multivariable regression model included age, race, sex, body mass index (BMI), susceptibility pattern of organism, MIC to eravacycline, immunocompromised, Charlson Comorbidity Index, hospital length of stay, ICU admission, vasopressor requirement, eravacycline treatment duration, concomitant antibiotics, and index culture site.

## RESULTS

### Demographics

A total of 48 patients receiving ≥72 h of eravacycline were included. Baseline demographic data are displayed in Table 1. The median age was 57.6 years, and most patients were female (60.4%, *n* = 29/48) and Black (54.2%, *n* = 26/48). The median (IQR) Charlson Comorbidity Index was 5.0 (3,7). Immunocompromised status was identified in 33.3% (*n* = 16/48) of patients, 27.1% (*n* = 13/48) being solid organ transplant patients on immunosuppression and 6.3% (3/48) immunosuppressed from chemotherapy.

**TABLE 1:** Baseline demographics

Variables	Treatment population (*n* = 48)	Treatment failure population (*n* = 19)	Treatment success population (*n* = 29)
Age, median (SD)	57.6 (15.4)	55.1 (15.9)	59.3 (15.2)
Females, *n* (%)	29 (60.4)	12 (63.2)	17 (58.6)
BMI, median (IQR)	29.9 (22.1, 36.4)	33.8 (23.9, 42.7)	27.4 (20.9, 29.4)
Race, *n* (%)			
Black	26 (54.2)	9 (47.4)	17 (58.6)
Caucasian	16 (33.3)	7 (36.8)	9 (31)
Pacific Islander	1 (2.1)	0 (0)	1 (3.4)
Unknown	5 (10.4)	3 (15.8)	2 (6.9)
Hispanic	2 (4.2)	1 (5.3)	1 (3.4)
CCI, median (IQR)	5 (3,7)	5.1 (3, 6.5)	5 (2,7)
Immunocompromised, *n* (%)	16 (33.3)	6 (31.5)	10 (34.5)
Solid organ transplant	13 (27.1)	5 (26.3)	8 (27.6)
Chemotherapy	3 (6.3)	1 (5.3)	2 (6.9)

### Clinical course

In total, 54.2% (*n* = 26/48) of patients were in the intensive care unit (ICU) and 41.7% (*n* = 20/48) of patients were on vasopressors at the time of index culture collection. Index culture specimens were most often isolated from the blood (27.1%, *n* = 13/48), respiratory tract (25%, *n* = 12/48), or wound(s) (22.9%, *n* = 11/48). Most patients, 91.7% (*n* = 44/48), received eravacycline dose of 1 mg/kg administered every 12 h. The median duration of eravacycline therapy was 12.6 (6, 13) days. Use of concomitant antimicrobial therapy within 72 h of initiation of eravacycline occurred in 77% (*n* = 37/48) of patients and most often consisted of vancomycin (12.5%, *n* = 6/37), micafungin (18.8%, *n* = 9/38), and/or meropenem (8.3%, *n* = 4/38). Clinical course data are shown in Table 2.

**TABLE 2:** Clinical course

Variables	Treatment population (*n* = 48)	Treatment failure population (*n* = 19)	Treatment success population (*n* = 29)
Hospital LOS, median (IQR)	56.9 (20.7, 51.5)	85.7 (25, 127)	67.3 (23, 61)
ICU admission, *n* (%)^*a*^	26 (54.2)	15 (78.9)	11 (37.9)
Vasopressor requirement, *n* (%)^*b*^	20 (41.7)	13 (68.4)	7 (24.1)
Culture specimen, *n* (%)			
Blood	13 (27.1)	6 (31.6)	7 (24.1)
Respiratory or sputum	12 (25)	7 (36.8)	5 (17.2)
Tissue or wound	11 (22.9)	3 (15.8)	8 (27.6)
Pleural or fluid	6 (12.5)	1 (5.3)	5 (17.2)
Urine	5 (10.4)	1 (5.3)	4 (13.8)
Other	1 (2.1)	1 (5.3)	0 (0)
Eravacycline treatment duration (IQR)	16 (33.3)	6 (31.5)	10 (34.5)
Eravacycline regimen, *n* (%)			
1 mg/kg every 12 h	44 (91.7)	16 (84.2)	28 (96.6)
1.5 mg/kg every 12 h	3 (6.3)	2 (10.5)	1 (3.4)
0.5 mg/kg every 12 h	1 (2.1)	1 (5.3)	0 (0)
Concomitant ABX^*c*^, *n* (%)	37 (77)	15 (79.2)	22 (75.9)
Adverse effects, *n* (%)	2 (4.2)	0 (0)	2 (6.9)
Diarrhea	1 (2.1)	0 (0)	1 (3.4)
Infusion site reaction	1 (2.1)	0 (0)	1 (3.4)

^*a*^ ICU admission at the time of index cultures.

^*b*^ Vasopressor requirement or mechanical ventilation within 24 h of index culture.

^*c*^ Concomitant antimicrobials most commonly were vancomycin, meropenem, micafungin, fluconazole, daptomycin, tobramycin, cefazolin, amikacin, and/or cefiderocol.

### Microbiological characteristics

Eravacycline was most often ordered as therapy to treat infections caused by VRE (31.3%, *n* = 15/48), *S. maltophilia* (29.2%, *n* = 14/48), and CRE (20.8%, *n* = 10/48). Eravacycline was also used for MDR Enterobacterales (14.6%, *n* = 7/48) and CRAB (12.5%, *n* = 6/48). Three patients received eravacycline for polymicrobial infections. (Table 3)

**TABLE 3:** Definitive eravacycline therapy

Organism, *n* (%)	Treatment population (*n* = 48)	Treatment failure population (*n* = 19)	Treatment success population (*n* = 29)
Vancomycin-resistant enterococci	12 (25)	5 (26.3)	7 (24.1)
Carbapenem-resistant *Acinetobacter* spp.	6 (12.5)	3 (15.8)	3 (10.3)
MDR Enterobacterales	5 (10.4)	1 (5.3)	4 (13.8)
*Klebsiella aerogenes*	2 (4.2)	1 (5.3)	2 (6.9)
*Klebsiella pneumoniae*	2 (4.2)	0 (0)	2 (6.9)
Carbapenem-resistant Enterobacterales	8 (16.7)	3 (15.8)	5 (10.3)
*Stenotrophomonas maltophilia*	14 (29.2)	7 (36.8)	7 (24.1)
Polymicrobial	3 (6.25)	0 (0)	3 (10.3)
VRE + CRE + MDR Enterobacterales	1 (2.1)	0 (0)	1 (3.4)
VRE + MDR Enterobacterales	1 (2.1)	0 (0)	1 (3.4)
VRE + CRE	1 (2.1)	0 (0)	1 (3.4)

### Clinical outcomes

**Fig 1:**
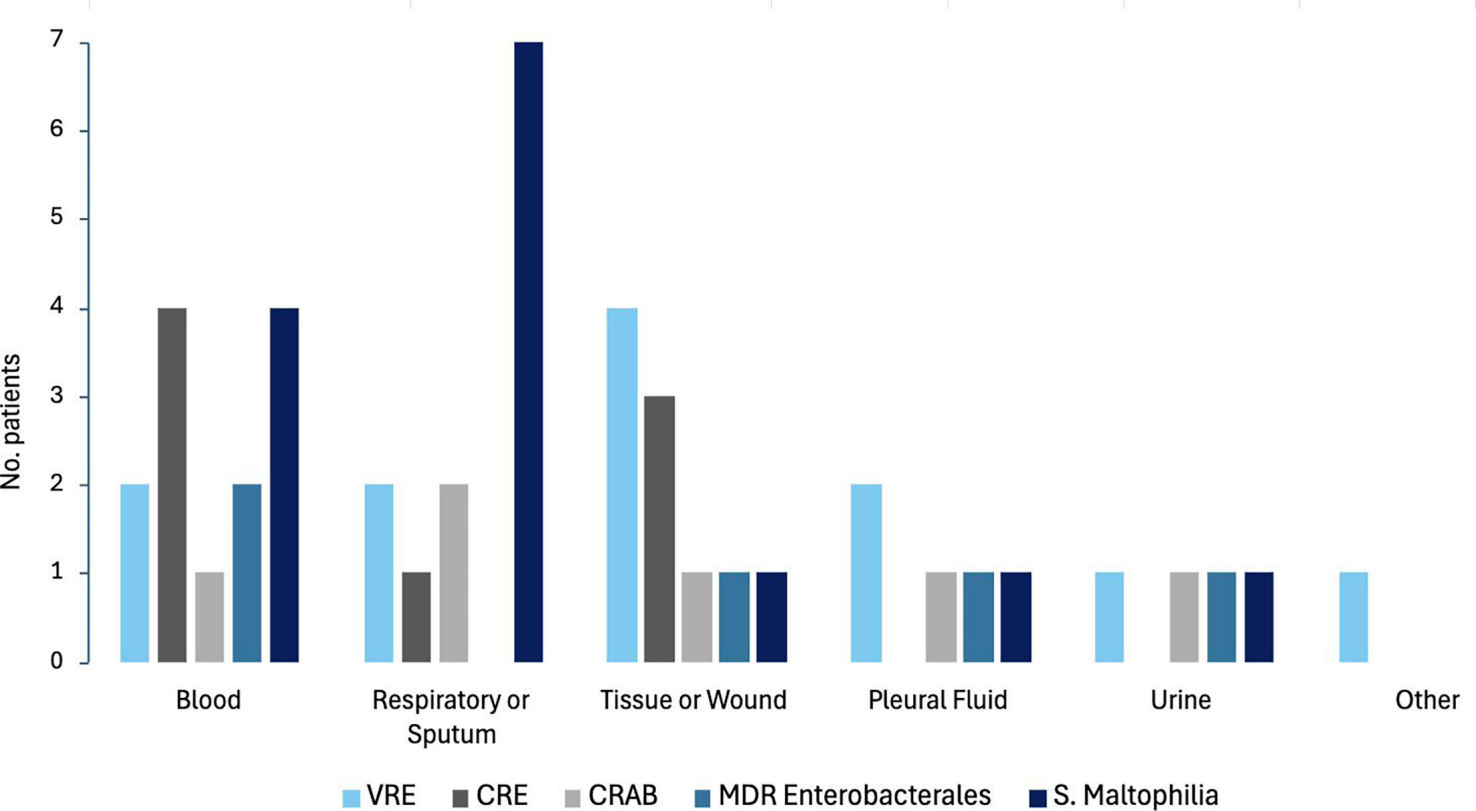
Specimen source and organism treated.

**Fig 2:**
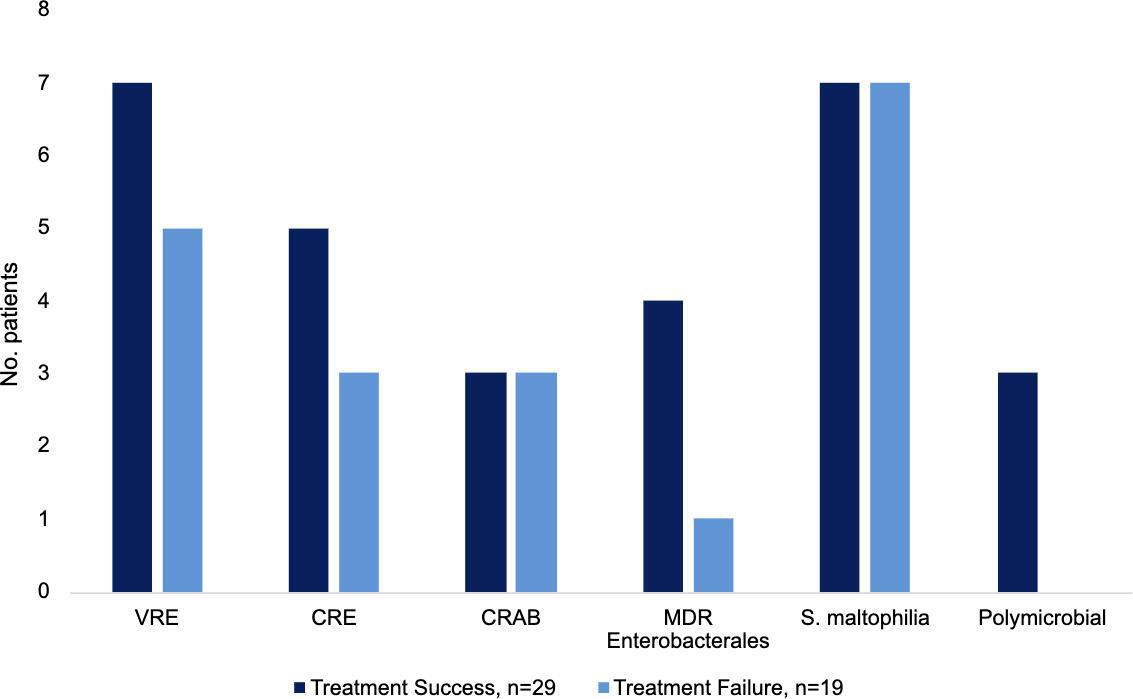
Organisms treated, with treatment failure and treatment success.

Overall, 60.4% (*n* = 29/48) of patients experienced survival and had no microbiological recurrence within 30 days after completing eravacycline. Patients who did not survive 30 days following eravacycline completion (39.6%, *n* = 19/48) had a positive respiratory (36.8%, *n* = 7/19) or blood (31.6%, *n* = 6/19) culture (Fig. 1). Eravacycline was associated with treatment success for 31% (*n* = 9/29) of infections due to VRE, 24.1% (*n* = 7/29) due to CRE, 24.1% (*n* = 7/29) due to *S. maltophilia,* 10.3% (*n* = 3/29) due to CRAB, and 13.8% (*n* = 4/29) due to MDR Enterobacterales, demonstrated in Fig. 2.

### Tolerability

Eravacycline adverse events occurred in 4.2% (*n* = 2/48) of patients with half being diarrhea (2.1%, *n* = 1/48) and the other half infusion site reactions (2.1%, *n* = 1/48). Overall, 2.1% (*n* = 1/48) of patients had eravacycline discontinued due to an adverse event, while 10.4% (*n* = 5/48) were switched to another agent, and 27.1% (*n* = 13/48) passed away during therapy with eravacycline.

### Multivariate analysis

The multivariate analysis found no difference between baseline characteristics and clinical course with treatment failure with eravacycline, displayed in Table 4.

**TABLE 4:** Multivariate analysis

Variables	*P*-value	CI 95%
Age, median (SD)	0.09	−0.58, 1.44
Females, *n* (%)	0.37	−0.36, 0.37
Race, *n* (%)	0.37	−0.05, 0.14
BMI, median (IQR)	0.55	−0.02, 0.01
CCI, median (IQR)	0.36	−0.10, 0.4
Immunocompromised, *n* (%)	0.71	−0.316, 0.46
Hospital LOS, median (IQR)	0.71	−0.003, 0.002
ICU admission^*a*^, *n* (%)	0.96	−0.57, 0.54
Vasopressor requirement^*b*^, *n* (%)	0.07	−1.19, 0.05
Eravacycline treatment duration (IQR)	0.47	−0.01, 0.02
Concomitant ABX^*c*^, *n* (%)	0.73	−0.32, 0.45
Index of culture site	0.46	−0.15, 0.07
Susceptibility	0.64	−0.12, 0.07

^*a*^ ICU admission at the time of index cultures.

^*b*^ Vasopressor requirement or mechanical ventilation within 24 h of index culture.

^*c*^ Concomitant antimicrobials most commonly were vancomycin, meropenem, micafungin, fluconazole, daptomycin, tobramycin, cefazolin, amikacin, and/or cefiderocol.

## DISCUSSION

This study evaluated the use of eravacycline for MDR infections, contributing to the growing body of literature supporting its broad-spectrum efficacy against MDR organisms. Eravacycline was primarily used as an adjunctive agent, most commonly for infections caused by VRE and *S. maltophilia*, despite lacking specific FDA indications for these infections. The majority of patients were critically ill, with 54.2% requiring intensive care and 41.7% on vasopressors at the time of treatment initiation. Most patients who had mortality within 30 days had a positive culture for *S. maltophilia* (36.8%) or VRE (26.3%). The multivariate analysis found no significant variables were associated with treatment failure with eravacycline due to lack of associations, suggesting that eravacycline’s effectiveness may not be easily predicted by baseline patient characteristics or clinical factors.

Eravacycline demonstrated a favorable safety profile, with a low incidence of adverse events. Only 2.1% of patients experienced gastrointestinal issues, and 2.1% had infusion site reactions, for an overall adverse event rate of 4.2%. This minimal side effect profile positions eravacycline as a promising treatment option for patients with limited alternatives due to its broad-spectrum activity and manageable safety profile.

The study aligns with previous research indicating that eravacycline’s broad-spectrum activity makes it a valuable agent of infections with limited therapeutic options. The findings in this study support previous reports highlighting eravacycline’s lower rate of adverse effects (4.2%) compared to other agents. Additionally, the low discontinuation rate due to side effects (2.1%) strengthens the evidence for eravacycline’s favorable tolerability profile. When comparing this study with the study by Kunz-Coyne and colleagues, we observe some differences in the clinical outcomes. In their study, clinical success occurred in 75.7% (*n* = 315/416) of patients with infections not further classified as caused by MDR organisms or other specific pathogens, while in our study, 60.4% (*n* = 29/48) of patients with MDR infections achieved clinical success (5). Notably, the definition of clinical success varied between the studies, with their criteria including survival, absence of microbiologic evidence at 30 days from the end of eravacycline therapy, and clinical improvement within 96 h of eravacycline initiation. Additionally, this study ran a multivariate regression analysis to look for clinical outcomes associated with treatment failure with eravacycline, which was not done in the Kuntz et al. study.

This difference could be attributed to a variety of factors, such as differences in the patient population. Our patient demographic differed from theirs in a few ways. The majority of our cohort was Black (54.2%), compared to only 29.3% in their study, where the majority of patients were Caucasian (56.7%) (5). Additionally, our patient population had a higher Charlson Comorbidity Index of 5, compared to 4.5 in the Kunz-Coyne study, suggesting that our patients may have a higher risk of mortality due to more comorbidities, which could have impacted the clinical success rates (5). Additionally, the Kunz-Coyne et al. study had a larger and more geographically diverse population across several medical centers, whereas our study was focused on a single health system.

Bacteremia was the most common infection for which eravacycline was used, shown in Table 2, despite tetracyclines generally being avoided in these cases due to their large volume of distribution. However, eravacycline was usually employed as a last-line agent for these MDR infections, often in combination with other therapies. Its use may have been intended for an unidentified infection source or to help reduce bacterial burden in the bloodstream. While tetracyclines are not typically preferred for bacteremia, studies have demonstrated eravacycline’s efficacy in such scenarios. Pooled data from the IGNITE 1 and IGNITE 4 studies showed that 87.5% of patients with concurrent bacteremia treated with eravacycline achieved clinical success, compared to 77% in the comparator group (16). These findings suggest that eravacycline may be a viable option for managing bacteremia, particularly in cases where other treatments have failed or are unsuitable.

Our study found higher mortality in patients with *S. maltophilia* treated with eravacycline compared to the other MDR infections. In general, *S. maltophilia* infections are associated with significant morbidity and mortality, with reported mortality rates ranging from 21% to 69% (17). Our study observed a mortality rate of 50% in patients with *S. maltophilia* (*n* = 7/14), which is within the typical range for this infection.

Our study offers valuable real-world evidence from a multi-center retrospective cohort within a large and diverse hospital network, encompassing both academic and community hospitals. By focusing on MDR infections, this study addresses the urgent need for alternative treatment options for these challenging infections. The inclusion of critically ill patients, many of whom were in the ICU and receiving vasopressors, provides a comprehensive evaluation of eravacycline’s effectiveness in high-risk clinical settings. Additionally, the use of multivariate regression analysis to identify predictors for treatment failure enhances the study. Despite not identifying significant predictors of treatment failure related to factors such as ICU status, Charlson Comorbidity Index, immunocompromised status, or infecting organism, these findings underscore the complexity of treatment outcomes in MDR infections.

There are several limitations to this study. As a retrospective chart review, it lacked standardization in treatment protocols and could not control for confounding variables including the use of other antimicrobials. Additionally, the absence of a control group makes it difficult to compare the efficacy of eravacycline with other treatment options. Moreover, since 77% of patients received concomitant antibiotics, it was challenging to isolate the effect of eravacycline alone on clinical outcomes.

In summary, eravacycline is being used for MDR infections and is tolerable, showing minimal side effects in our patient population. Eravacycline is a well-tolerated, broad-spectrum agent that we found to be reasonable to use for the treatment of MDR infections at this health system. Overall, eravacycline appears to be an effective option for treating severe infections due to MDR pathogens in critically ill patients, with a manageable safety profile. Further studies are needed to evaluate its long-term efficacy, especially in high-risk populations with persistent or resistant infections.
